# Data for rapid ethanol production at elevated temperatures by engineered thermotolerant *Kluyveromyces marxianus* via the NADP(H)-preferring xylose reductase–xylitol dehydrogenase pathway

**DOI:** 10.1016/j.dib.2015.08.038

**Published:** 2015-09-09

**Authors:** Biao Zhang, Jia Zhang, Dongmei Wang, Xiaolian Gao, Lianhong Sun, Jiong Hong

**Affiliations:** a School of Life Science, University of Science and Technology of China, Hefei, Anhui 230027, PR China; b Department of Biology and Biochemistry, University of Houston, Houston, TX 77004-5001, USA; c Hefei National Laboratory for Physical Science at the Microscale, Hefei, Anhui 230026, PR China

**Keywords:** *Kluyveromyces marxianus*, Xylose, Ethanol, Co-assimilation, Elevated temperature

## Abstract

A thermo-tolerant NADP(H)-preferring xylose pathway was constructed in *Kluyveromyces marxianus* for ethanol production with xylose at elevated temperatures (Zhang et al., 2015 [25]). Ethanol production yield and efficiency was enhanced by pathway engineering in the engineered strains. The constructed strain, YZJ088, has the ability to co-ferment glucose and xylose for ethanol and xylitol production, which is a critical step toward enabling economic biofuel production from lignocellulosic biomass. This study contains the fermentation results of strains using the metabolic pathway engineering procedure. The ethanol-producing abilities of various yeast strains under various conditions were compared, and strain YZJ088 showed the highest production and fastest productivity at elevated temperatures. The YZJ088 xylose fermentation results indicate that it fermented well with xylose at either low or high inoculum size. When fermented with an initial cell concentration of OD_600_=15 at 37 °C, YZJ088 consumed 200 g/L xylose and produced 60.07 g/L ethanol; when the initial cell concentration was OD_600_=1 at 37 °C, YZJ088 consumed 98.96 g/L xylose and produced 33.55 g/L ethanol with a productivity of 0.47 g/L/h. When fermented with 100 g/L xylose at 42 °C, YZJ088 produced 30.99 g/L ethanol with a productivity of 0.65 g/L/h, which was higher than that produced at 37 °C.

**Specifications table**

**Table:** 

Subject area	Biology
More specific subject area	Xylose metabolism
Type of data	Table; figure
How data was acquired	The metabolic products were acquired by HPLC using an Agilent 1100 series HPLC system. XR and XDH activity were determined using a spectrophotometer to monitor the change in A340 upon oxidation of NAD(P)H.
Data format	Raw and analyzed
Experimental factors	No pretreatment
Experimental features	Batch fermentation; HPLC; enzyme activity
Data source location	Not applicable
Data accessibility	The data are supplied with this article.

**The value of the data**

● Comparison of the fermentation results of the different engineered strains during pathway engineering revealed the specific role of genes related to xylose metabolism under oxygen-limited conditions.
● Compared with other reported yeast strains, *K. marxianus* YZJ088 showed considerable ethanol production and the highest ethanol productivity.
● Strain YZJ088 fermented xylose well with an initial OD=1 or 15 at 37 °C, which indicates this strain produced more ethanol with relative lower productivity [25].
● *K. marxianus* YZJ088 fermented xylose well with an initial OD=1 at 42 °C, and the co-fermentation of glucose and xylose indicates that it has great potential for application in simultaneous saccharification and fermentation at elevated temperatures.
● Though it produced relative less ethanol, the productivity of YZJ088 at 42 °C was faster.

## 1 Data, experimental design, materials and methods

### 1.1 Comparison of the xylose fermentation ability of constructed strains

To compare the effects of over-expression or disruption of downstream genes, *K*. *marxianus* strains YZJ020, YZJ051, YZJ061, YZJ069, YZJ071, YZJ077, YZJ084, YZJ086, YZJ088, YZJ089, and YZJ091 (Table 2 in Ref. [25]), which were constructed during pathway engineering, were fermented with YP medium that contained 100 g/L xylose at 42 °C with 250 rpm and initial OD_600_=15 under oxygen-limited conditions [25]. The over-expression of genes involved in xylose metabolic promoted ethanol production in the engineered strains (Table 1). *KmFPS*1 disruption reduced xylitol accumulation and utilization but blocked the production of glycerol (Table 1).

### 1.2 Comparison of ethanol producing abilities from xylose with various previously reported yeast strains

The ethanol-producing ability with xylose at 42 °C of *K. marxianus* YZJ088 was compared with other ethanol fermentation yeast strains. *K. marxianus* YZJ088 exhibited considerable ethanol production and the highest ethanol productivity at elevated temperatures (Table 2).

### 1.3 *K. marxianus* YZJ088 fermented well with a high concentration xylose at 37 °C

**Fig. 1:**
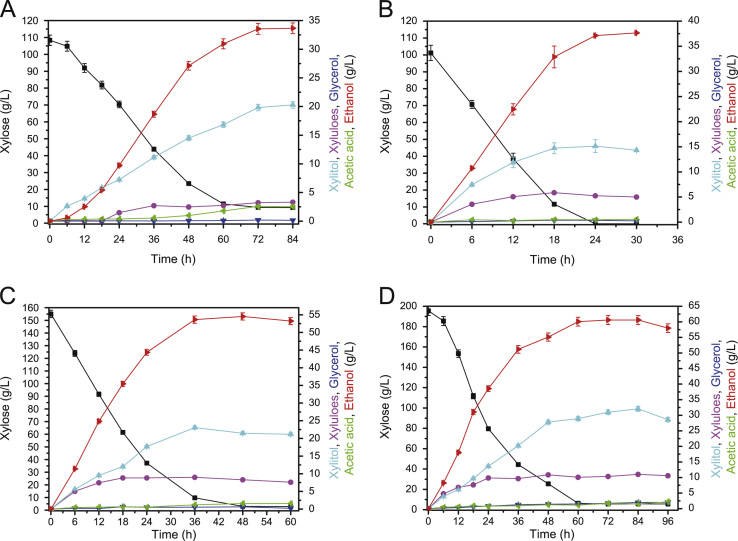
Fermentation of YZJ088 in YP medium at 37 °C with 100 g/L xylose and initial OD_600_=1 (A), 100 g/L xylose and initial OD_600_=15 (B), 150 g/L xylose and initial OD_600_=15 (C), 200 g/L xylose and initial OD_600_=15 (D). The values are the means of three biological replicates±standard deviation (*n*=3).

The fermentation ability of *K. marxianus* YZJ088 at 37 °C was explored. *K. marxianus* YZJ088 fermented 100 g/L xylose and produced 33.55 g/L ethanol in 72 h with an initial OD_600_=1. When increased to an initial OD_600_=15, YZJ088 could ferment 100, 150, and 200 g/L xylose and produced 37.13, 53.62, and 60.07 g/L ethanol with productivities of 1.55, 1.49, and 1.00 g/L/h, respectively (Fig. 1). Although YZJ088 used more xylose and produced more ethanol at 37 °C, faster productivity (2.49 g/l/h) was achieved at 42 °C (Table 1 and Fig. 1) [25].

### 1.4 *K. marxianus* YZJ088 fermented xylose at 42 °C with low inoculum size

**Fig. 2:**
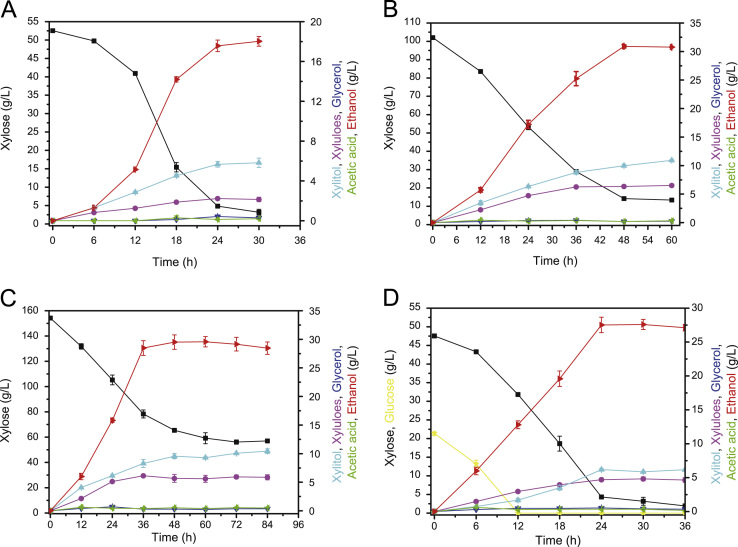
Fermentation of YZJ088 in YP medium with 50 g/L xylose (A), 100 g/L xylose (B), 150 g/L xylose (C) and 50 g/L xylose+20 g/L glucose (D) at 42 °C with initial OD_600_=1. The values are the means of three biological replicates±standard deviation (*n*=3).

*K. marxianus* YZJ088 fermented 50, 100, 150 g/L xylose, and a 20 g/L glucose-50 g/L xylose mixture with an initial cell concentration of OD_600_=1 at 42 °C under oxygen-limited conditions and produced 18.03, 30.99, 28.48, and 27.52 g/L ethanol, respectively (Fig. 2). Although most xylose fermentation was conducted at high inoculum size, YZJ088 produced ethanol fairly well at 42 °C with low inoculum size. However, when xylose concentration reached 150 g/L, the ethanol production was limited. These results may have occurred because xylose tolerance decreased at higher temperatures [23,24].

### 1.5 XR and XDH activities of *K. marxianus* strains growth at 37 °C were higher than those at 42 °C

XR and XDH activities were determined for NBRC1777, YZJ020, YZJ051, and YZJ088 cells cultured with YP medium contained 20 g/L xylose at 37 °C. The cells were harvested by centrifugation at 10,000×*g* for 10 min at room temperature and washed with 100 mM potassium phosphate buffer (pH 7.4). The cells were resuspended in the same buffer and then lysed by sonication (Vibra-Cell VC505, Connecticut, USA) for 20 min at 40% power in an ice–water bath. The cell debris was removed by centrifugation at 10,000×*g* for 10 min, and the supernatant was used to measure enzyme activity. The assay mixture (1.0 mL) for the XR enzyme reaction contained 100 mM of phosphate buffer (pH 7.4), 200 μM NAD(P)H, 200 mM xylose, and crude enzyme solution (0.1 mL). The assay mixture (1.0 mL) for the XDH enzyme reaction contained 50 mM MgCl_2_, 50 mM Tris–HCl buffer (pH 9.0), 20 mM NAD(P)^+^, 300 mM xylitol, and crude enzyme solution (0.1 mL). The reaction was started by adding 0.1 mL of crude enzyme. One unit of enzyme activity is defined as the amount of enzyme required to oxidize/reduce 1 μmol of NAD(P)H/NAD(P)^+^ per min under the specified conditions [22].

**Fig. 3:**
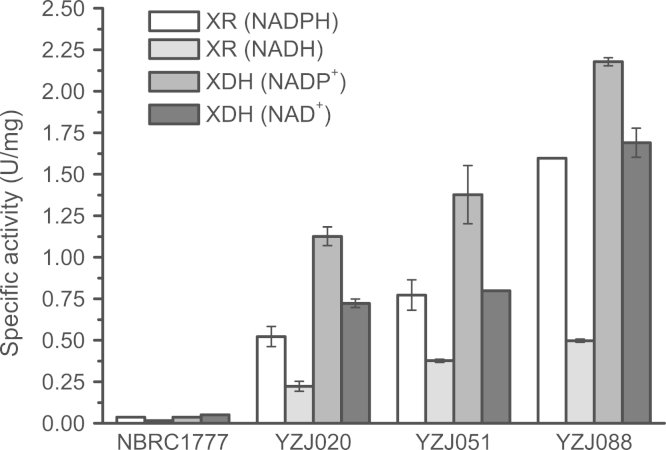
Comparison of the XR and XDH activities of NBRC1777, YZJ020, YZJ051 and YZJ088 cultured in YP medium with 20 g/L xylose at 37 °C for 24 h. The values are the means of three biological replicates±standard deviation (*n*=3).

XR and XDH activities in these strains growth at 37 °C were higher than those at 42 °C (Fig. 3) [25]. The XR (NADPH) and XDH (NADP^+^) activities of YZJ088 cultured at 37 °C were 3.69- and 3.91-fold higher, respectively, than those at 42 °C. Although the enzymatic activities at 37 °C were higher than those at 42 °C, they did not yield higher productivity. More xylitol accumulation at 37 °C with high xylose concentration could reflect lower efficiency of the downstream enzymes at 37 °C [25].

## Figures and Tables

**Table 1:** Summary of the fermentation by engineered strains with YP medium containing 100 g/L xylose at 42 °C.

Strains	Time (h)	Residual xylose (g/L)	Xylulose (g/L)	Xylitol (g/L)	Glycerol (g/L)	Acetate (g/L)	Ethanol (g/L)	Ethanol productivity (g/L/h)

YZJ020	18	21.14±1.25	2.8±0.69	10.78±1.02	5.56±1.54	1.06±0.52	25.48±0.57	1.42±0.24
YZJ051	18	16.49±0.96	3.72±0.58	9.21±2.30	5.53±1.44	1.22±0.34	29.73±1.24	1.65±0.52
YZJ061	18	12.2±1.56	3.9±1.34	10.29±2.11	6.84±1.63	1.34±0.40	31.99±2.31	1.78±0.30
YZJ077	18	10.4±1.50	3.97±0.32	9.46±2.13	6.48±1.52	1.24±0.33	31.38±1.47	1.74±0.41
YZJ084	18	11.6±2.41	1.88±0.64	4.80±1.02	6.70±2.01	0.98±0.29	33.90±1.38	1.88±0.74
YZJ086	18	6.57±1.63	9.13±1.61	12.25±2.52	0.13±0.03	0.48±0.31	33.78±1.29	1.88±0.50
YZJ088	18	3.9±0.96	9.00±1.85	11.86±3.44	0.15±0.04	0.70±0.41	35.94±1.24	2.00±0.34
YZJ089	18	3.82±1.32	9.27±2.41	11.94±2.12	0.91±0.32	0.67±0.28	34.36±0.98	1.91±0.69
YZJ091	18	5.39±1.21	5.11±2.31	8.32±2.84	0.19±0.08	0.62±0.19	33.21±2.07	1.85±0.34

**Table2:** Comparison of the xylose consumption and the ethanol production among the various yeast strains^a^.

Strains	Temperature (°C)	Xylose (g/L)	Initial OD	Xylose consumption (g/L)	Xylitol production (g/L)	Xylitol yield (g/g)	Ethanol production (g/L)	Ethanol yield (g/g)	Ethanol productivity (g/L/h)	Time of fermentation (h)	Reference
*K. marxianus* SUB-80- S	35	20	1/20 volume	20	NR	NR	5.6	0.28	0.12	48	[12]
*K. marxianus* IMB4	40	20	0.22 g/L	13.61	7.36	0.54	2.08	0.15	0.022	96	[19]
*K. marxianus* DMKU3-1042	40	20	OD600=1	20	~6.5	~0.33	2.2±0.2	0.11±0.01	0.046±0.001	48	[15]
*Kluyveromyces* sp. IIPE453	50	20	OD600=1	~17.5	11.5±0.4	0.66±0.02	1.75±0.05	0.10±0.01	0.025±0.001	80	[9]
*K. marxianus* YZB014	42	20	OD600=10	19.00±1.00	11.32±0.36	0.60±0.02	3.55±0.19	0.19±0.01	0.110±0.006	32	[21]
*K. marxianus* YRL002	42	50	OD600=10	30.15	–	–	11.52	0.38	0.069	168	[17]
*H. polymorpha* CBS4732	48	120	OD600~5	~16	0.02	0.00125	1.31	0.08	0.054	24	[2]
*H. polymorpha* 2EthOH^−^ /XYL1m/XYL2/XYL3/BrPA	45	92	2 g/L	32.67	0	0	9.8	0.3	0.18	55	[10]
*S. cerevisiae* SXA-R2P-E	30	40	OD600=20	36.67	–	–	16.5	0.45	0.28	60	[11]
*S. cerevisiae* PUA6-9	30	20	1/10 volume	19.65	9.88	0.50	3.08	0.16	0.04	76	[8]
*S. cerevisiae* TMB 3057	30	50	OD600=10	39.6±3.4	8.71±1.19	0.22±0.03	13.30±1.70	0.33±0.02	0.133±0.017	100	[4]
*S. cerevisiae* F106KR	30	165	OD600=10	161.2	20.6	0.13	58.5	0.36	1.22	48	[20]
*S. cerevisiae* F106KR	30	221.1	OD600=10	212.0	21.7	0.10	77.6	0.37	1.08	72	[20]
*S. cerevisiae* DA24-16	30	80	4 g/L	79.7	3.2	0.04	27.9	0.35	0.47	60	[3]
*S. cerevisiae* CIBTS0735	30	40	OD600=10	39.7	–	–	17.47	0.44	1.09	16	[1]
*S. cerevisiae* DGX23	30	40	OD600=1.3	32.28	2.00	0.06	9.36	0.29	0.13	72	[7]
*S. cerevisiae* Y-ARSdR^b^	30	15^c^	NR	13.6	4.00	0.29	7.02	0.46	0.10	72	[18]
*S. cerevisiae* MA-N5^b^	30	45	OD600=15	40.56	2.64	0.07	14.6	0.36	0.20	72	[13]
*S. cerevisiae* D-XR/XDH/XK^b^	30	15^c^	OD600=10	12.75	2.74	0.21	8.00	0.43	0.11	72	[14]
*S. cerevisiae* SK-N2^b^	30	55^d^	NR	~55	~3.8	0.07	30.1	0.41	0.18	168	[6]
*S. cerevisiae* SK-NN^b^	30	20	NR	15	~4.4	0.29	4.02	0.27	0.03	144	[5]
*S. passalidarum* NN245	25	150	OD600=15	150	–	–	53.3	0.36	0.44	120	[16]
*K. marxianus* YZJ088	42	128.46±3.91	OD600=15	118.39±2.91	11.09±1.47	0.09±0.01	44.95±3.21	0.38±0.02	2.49±0.18	18	This study

NR: not reported.

a If the literature described several strains, only the best one is shown.

b Strains with NADPH-NADP^+^ xylose metabolic pathway.

c Fermentation with 5 g/L glucose as co-substrate.

d Fermentation with 20 g/L glucose as co-substrate.
